# Detection of Borna Disease Virus (BDV) in Patients with First Episode of Schizophrenia

**Published:** 2016-10

**Authors:** Hasan Soltani, Serwa Mohammadzadeh, Manoochehr Makvandi, Siroos Pakseresht, Alireza Samarbaf-Zadeh

**Affiliations:** 1 Health Research Institute, Infectious and Tropical Diseases Research Center, Ahvaz Jundishapur University of Medical Sciences, Ahvaz, Iran.; 2 Department of Psychology, Faculty of Economics & Social Sciences, Bu-Ali Sina University, Hamedan, Iran.; 3 Department of Virology, Ahvaz Jundishapur University of Medical Sciences, Ahvaz, Iran.; 4 Department of Psychiatry, Golestan Hospital, Ahvaz Jundishapur University of Medical Sciences, Ahvaz, Iran.; 5 Department of Pathology, Health Research Institute, Infectious and Tropical Diseases Research Center, Ahvaz Jundishapur University of Medical Sciences, Ahvaz, Iran.

**Keywords:** *BDV*, *Nested RT-PCR*, *Schizophrenia*

## Abstract

**Objective:** Schizophrenia is a complex widespread neuropsychiatric disorder. This illness encompasses a complex debilitating mental disorder causing illusion, delusion, disturbed relationship, low motivation and decline of emotion. Viral infection of the brain including Borna Disease Virus (BDV) may play a role in transient or permanent neurological and behavioral abnormalities. This role of Borna virus has not been resolved outright yet, and based on published papers investigation examining the role of this virus in schizophrenia is in progress worldwide.

**Method:** In this study, Nested Reverse Transcription–Polymerase Chain Reaction (Nested RT-PCR) was used for detection of BDV Ribonucleic Acid (RNA) in Peripheral Blood Mononuclear Cells (PBMCs) of a group of patients experiencing the first episode of schizophrenia. The results were compared with a normal group.

**Results:** In our study, no BDV-positive was found in PBMCs of the case group. Out of 40 participants of control group one was positive for P24 gene of BDV. This result are similar to several published papers about this topic.

**Conclusion**: An etiological relationship between Bornavirus and schizophrenia was not found in this study. More investigations are warranted to illustrate the probable relationship between bornavirus infection and schizophrenia.

Schizophrenia is a complex widespread neuropsychiatric disease (1). This abnormality encompasses a complex debilitating mental disorder causing illusion, delusion, disturbed relationship, low motivation and decline of emotion decline (2). Treatment of schizophrenia that usually starts at early adulthood is costly in all over the World. Prevalence of this illness is equal in both genders, but its progression is not similar with milder outcome in females (3).

The etiology of schizophrenia is idiopathic. Based on the collected data from epidemiological studies of families and identical twins, there is a very complicated interaction between genetic and environmental risk factors (including viral agents) on the maturation of the brain predispose, causing some people to develop Schizophrenia (1). According to a cohort study performed in Finland, presence of some viruses in the CNS of infants enhanced the chance of schizophrenia in their adulthood by a factor of 5 (4).

Viral infection of the brain including Borna Disease Virus (BDV) can lead to transient or permanent neurological and behavioral abnormalities. Therefore, the study of the role of BDV in schizophrenia has increased in the past 20 years (5). 

BDV, a member of the order of Mononegavirales, is an enveloped non-segmented negative single-stranded RNA virus (6, 7). This virus probably infects its host through olfactory route, capable of replicating in the neurons, astrocytes, Schwan cells and oligodendroglia, (8). A wide range of warm-blooded animals including human, domestic and exotic mammals host BDV. Infection of animals inserts changes in the brain tissue that may lead to behavioral abnormalities and movement difficulties, sometimes with psychological manifestations similar to schizophrenia (9).

The severity of BDV infection is dependent on age, immune status, genetic and maturity of the development of CNS of the host (1). Involvement of BDV in schizophrenia is based on seroepidemiology, detection of BDV RNA in Peripheral Blood Mononuclear Cells (PBMCs) and autopsy of brain tissue of schizophrenic patients (1, 8- 10). Since BDV can persist in the brain cells, the role of this virus in schizophrenia, bipolar and autism is not out of the question (11).

Therefore, the authors decided to conduct a research for a re-evaluation of the role of BDV in schizophrenic patients in Ahvaz, Iran. In this study, the results were compared with a healthy control group.

## Materials and Method

This case-control study was conducted to survey the prevalence of BDV infection between two groups: a group with the first episode of schizophrenia (FESP), and a group of normal participants in Ahvaz South-west Iran.

Before taking the specimens, the normal group and legal guardians of the patients were informed about the aim of the study and a consent form was obtained and delivered to the psychologist of FESP and normal groups. Information such as address, age, gender, marital status, profession, history of previous mental disorder and confounding factors (history of alcohol consumption, addiction, keeping pets, living in a village) were collected in a questionnaire for each participant. The Ethical Committee of Jundishapur University of Medical Sciences in Ahvaz approved the study.

***Selection of FESP Group***

During 8 months, 40 FESP (5 females and 35 males), with the mean age of 33.45, a minimum of 19.05, and maximum 37.03 participated in this study from four teaching hospitals in Ahvaz, which have psychiatric wards. Members of FESP group were diagnosed as schizophrenic cases for the first time (based on legal guardian patients’ information in the questionnaire and psychiatrists’ interview) and naïve to all specific drugs for schizophrenia. Criteria for including the clinical group was the final diagnosis of schizophrenia based on DSM-IV (Diagnostic and Statistical Manual of Mental Disorders, 4th Edition, Text Revision) by the residents of the psychiatry hospitals. To ensure diagnostic adequacy, MMPI test was filled for this group, and they received the cutoff point of schizophrenia diagnosis in clinical subscales (2, 7, and 8) of MMPI. Members of this group were not suffering from any disease affecting their immune status. Their HBs Antigen, HCV and HIV antibodies, the level of Serum c-Reactive Protein (SCRP) and Serum Total Protein (STP) were checked. The participants should not have a history of drug abuse and consumption of alcohol or an acute infection for at least two weeks before sampling. 

***Selection of the Normal Group***

Forty individuals whose age and gender were matched with the members of FESP group were selected randomly. Based on Minnesota Multiphasic Personality Inventory, none of the participants of the control group had schizophrenia by these criteri. The questionnaire and checklist were filled up under the supervision of the psychologist of our group. Other criteria for the test group such as immunological status etc. were considered for the control group as well.

***Phlebotomy (Blood Extraction)***

Three-milliliter blood was collected in a tube without anti-coagulant for serology and biochemistry tests. Another 4-milliliter blood was mixed with EDTA for separation of PBMCs. The tubes of the samples were numbered and labeled randomly. To avoid the risk of contamination, the process of handling and testing the cases and control group were performed in separate lab-rooms. 

***Preparation of Sera for Serology and Biochemistry***

Sera of coagulated blood tubes were collected and subjected to STP (Pars Azmun, Iran). To evaluate the immune system status of the participants, the Biuret method and auto-analyzer (Roche/Hitachi 902 Japan) were used. Internal and external quality control was used in the test. STP of all members was in the normal range (FESP group: 6.72 g/dL & control group: 6.57 g/dL). HBs Antigen, HIV and HCV antibody of all participants were negative except for a member of the control group who was HBsAg positive and was omitted from the study and replaced by a healthy HBs antigen negative sample. To rule out acute infection, SCRP was measured by auto-analyzer (Roche/Hitachi 902. JAPAN. Bionik Kit, IRAN) two weeks before sampling. The figures were in the normal range for all samples.

***Preparation of Peripheral Blood Mononuclear Cells (PBMNCs)***

PBMCs of the test and control group samples were separated immediately from 4 ml anti-coagulated blood by Lymphocyte Separated Ficoll (Baharafshan, Iran). The number of PBMCs for each sample was calculated by a hemocytometer slide.

RNA Extraction:

RNA was extracted from PBMCs by AccZol (Bioneer kit, South Korea) according to the manufacturer’s instruction to be used as templates for preparation of their cDNAs. 

***Synthesis of Complementary DNA (cDNA)***

Concentration, purity and integrity of extracted RNA were calculated and checked by 260 and 280 nm UV-spectroscopy, and gel electrophoresis of 2 µl of RNA. Then 5 µl of RNA was used as the template for cDNA preparation. The prepared cDNA was used as the template of Nested- PCR.

***Nested Polymerase Chain Reaction (Nested PCR)***

**Figure1:**
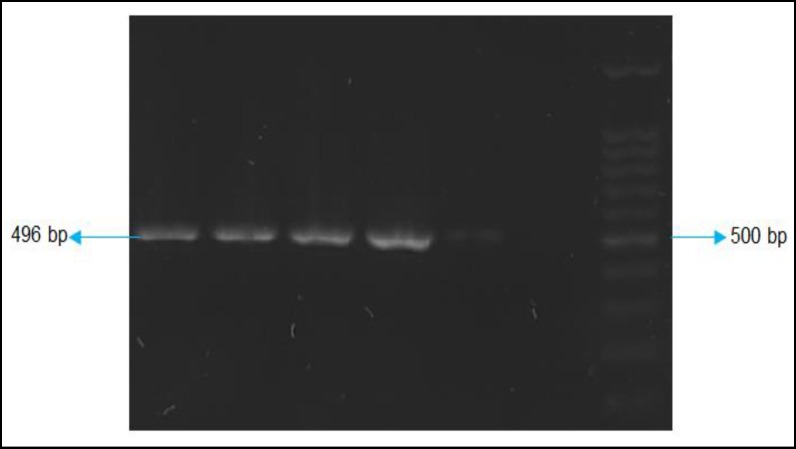
Gel Electrophoresis of PCR Products for GAPDH. The 100 bp DNA Size Marker Is Shown on the Right of the Figure

For amplification of gene P24 of BDV, the external and internal primers were synthesized according to Kishi et.al (12). PCR program for both first and the second rounds was similar, except that in the second round annealing Tm was 55 ˚C (table 1). For each round of PCR, negative controls (H2O and RNA), and positive controls (GAPDH, P24 synthetic DNA) were included in each set of reactions (Figure 1).

***Gel Electrophoresis***

Ten milliliters of PCR product was loaded onto 1.4% agarose gel containing 1% DNA safe-stain and subjected to 100 volts electrophoresis (CinaGen Co. Iran). A 392 fragment was expected to be visible for P24 of BDV and 496bp for GAPDH reaction.

***Statistical analysis***

Chi-Square and t -test were used for data analysis, using SPSS software Version 15.

## Results

***Demographic Data***

Table 2 demonstrates the demographic data of the participants of the test and control groups. This table displays no significant difference between the two groups in terms of age (p-value = 1.00) and gender (p-value = 1.00). However, there was a significant difference in terms of marital status (p-value = 0.00) and care of animals (p-value = 0.003) between the two groups. 

***Molecular Test Results***

**Figure2:**
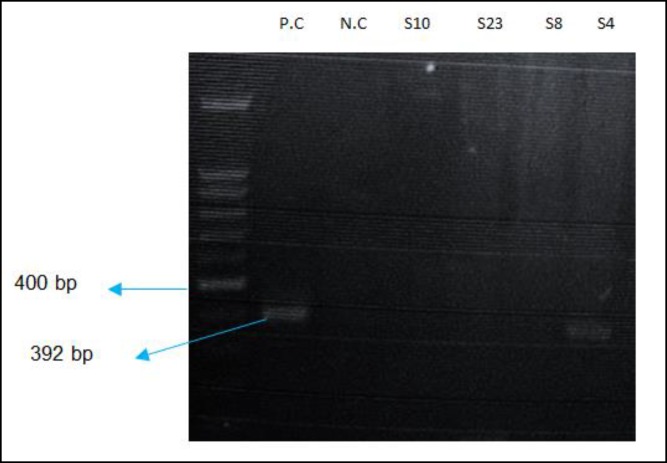
Gel Electrophoresis of Nested-PCR Products for P24 of BDV/P.C: Positive Control/ N.C: Negative Control/ S: Sample. S4 Is the Positive Sample. A 100 bp DNA Molecular Marker Was Used as a DNA Ruler

In this study, no positive case was found in PBMCs of the case group. Out of 40 participants of the control group, one was positive for P24 of BDV (Figure 2).

## Discussion

The association between BDV infection and schizophrenia is not well established. Reports about the role of BDV as a risk factor involving in this illness are highly contradictive. Previous, movement and behavioral abnormality in animals following BDV infection were observed. Nevertheless, the role of infection with this virus was ambiguous in schizophrenia. The aim of this study was to evaluate the role of BDV infection in schizophrenic patients in Ahvaz, Iran.

Early epidemiological studies on mentally disordered cases and normal blood donors to find a link between BDV infection and schizophrenia were not fully decisive. For example, Nakamura and colleagues studied the autopsy of brain tissue of four schizophrenic patients and two healthy individuals as a control group. They found RNA of BDV in the brain tissue of a schizophrenic patient by Nested RT-PCR. In addition, the serum of the case was positive for P24 Ag of BDV (10).

**Table1:** Temperature, Time and Number of Cycles in Different Steps of PCR for Amplification of Fragments

**STEP**	**Temperature(**ºC**)**	**Time (min or second)**	**Number of Cycles**
**Initial Denaturation**	95	5 (min)	1 (Firstly)
**Denaturation**	95	30 (second)	35
**Annealing**	53 (55)	30 (second)	35
**Extension**	72	30 (second)	35
**Finally Extension**	72	10 (min)	1 (Finally)

**Table2:** Demographic Data of the FESP and Normal Groups Are Showed

Demographic Data	FESP Group	Normal Group	p-Value
**Female/male (total)**	**5 / 35 (40)**	**5 / 35 (40)**	**1.00**
**Mean age (minimum/maximum)** **Total** **Female** **Male**	**33.5 (19.05 / 57.03)** **36.8 (20.01 / 57.03)** **33 (19.05 / 53.02)**	**33.5 (19.05 / 57.03)** **36.8 (20.01 / 57.03)** **33 (19.05 / 53.02)**	**1.00**
**Marital (frequency / percent)** **Single** **Married** **Divorced**	**(33 / 82.5)** **(6 / 15)** **(1 / 2.5)**	**(10 / 25)** **(30 / 75)** **0**	**0.00**
**Care of Animal** **(Frequency / percent)** **No** **Yes**	**(9 / 22.5)** **(31 / 77.5)**	**(23 / 57.5)** **(17 / 42.5)**	**0.003**

Puerto and colleagues studied 70 schizophrenic cases and 70 healthy subjects. Western – blot of their sera showed that 21.43% of the schizophrenic cases had BDV -P24 antibody, while sera of all of the control group members lacked this antibody; the difference was statistically significant between the two groups (P<0.0001) (13).

In Brazil, PBMCs of 27 schizophrenic patients were searched for BDV RNA; of them, 12 (44.4%) were positive for BDV RNA, whereas 14.8% of the control group were positive for this virus (1).

Zhang and colleagues studied PBMCs of 81 schizophrenic patients for BDV RNA with RT-quantitative PCR and compared the results with healthy blood donors. While less than 1% of blood donors were positive for BDV RNA, 9.9% of the cases were BDV RNA positive (14).

In Tehran (Iran), the technique of Circulatory Immune Complex (CIC) was applied for the study of BDV in schizophrenic cases compared to healthy subjects. The figures were 29.5% and 40.4% for healthy subjects and schizophrenic cases alternatively (15).

However, there are many reports that do not show any correlation between BDV infection and schizophrenia. Iwata and colleagues, in Japan, reported that 2% of blood donors and 4% of schizophrenic cases were BDV positive (16). In a paper published by Selten and colleagues, the prevalence of BDV in the control group was more than in schizophrenic patients in the Netherlands (17), whose result was similar to our study. In addition, NA and colleagues, in Korea did not find BDV in PMBCs of normal participants and schizophrenic cases (18). 

To the best of our knowledge, our study was the first attempt of this kind to be performed in Ahvaz, Southwest Iran. Usually, the prevalence of BDV in residents of a geographical area shows a correlation with the infected animal reservoir of the area with this virus. Bahmani and colleagues conducted an investigation in Tehran on BDV infection of various horse species. Considering the result of Mazaheri-Tehrani and report of Bahmani and colleagues, it is suggested that BDV infection of humans and horses of the same geographical area correlate with each other (15, 19).

Although high percentages of participants of case and control groups in our study had a background of dealing with domestic animals, only one sample of the normal group was positive for BDV. 

Altogether, we could not emphasize BDV as a risk factor to detect schizophrenia in Ahvaz patients. Thus, one possibility is that this kind of virus is not widespread in this region of Iran.

The important point to note is that in the most of the previous studies on this subject, the patients under study were held for a long time in mental hospitals, or they had the record of several hospitalizations. However , in order to exclude the confounding factors such as: (1- decreased hygiene level of schizophrenic patients during illness; 2- increased contacts with the animals of some patients during illness; 3- infection of schizophrenic patients with BDV during hospitalizations.), in this study, only FESP group was included. We believe that this kind of studies must be conducted on FESP patients to reflect the actual BDV prevalence in schizophrenic patients. Therefore, concerning the conflicting results from different studies, further epidemiological and molecular investigations are warranted to evaluate the probable BDV effect as an environmental risk factor. The following considerations are suggested for future studies: Including FESP in the study group; and the animals of the residential area of schizophrenic patients in the sense of infection with BDV virus should be examined.

## Limitations

Some patients or their families were not collaborative.

## Conclusion

We believe that this kind of studies must be conducted on FESP patients to reflect the actual BDV prevalence in schizophrenic patients. Therefore, concerning the conflicting results from different studies, further epidemiological and molecular investigations are warranted to evaluate the probable BDV effect as an environmental risk factor. The following considerations are suggested for future studies: Including FESP in the study group; and the animals of the residential area of schizophrenic patients in the sense of infection with BDV virus should be examined.
